# Deciphering the Regulatory Network of Tail Fat Deposition in Large- and Small-Tailed Han Sheep Through Transcriptome and MicroRNAome Profiling

**DOI:** 10.3390/biology15020179

**Published:** 2026-01-19

**Authors:** Guan Wang, Liming Tian, Shuhong Zhang, Zhaohua He, Fangfang Zhao, Menghan Chang, Wei Han, Dandan Ye, Jingyi Gao, Shaobin Li, Guangli Yang

**Affiliations:** 1 College of Biology and Food, Shangqiu Normal University, Shangqiu 476000, China; 13680740a@sina.com (G.W.); liming202506@163.com (L.T.); shuhongzhang_2013@163.com (S.Z.); hezh1250148718@163.com (Z.H.); changmenghan1005@163.com (M.C.); hanwei00005@163.com (W.H.); 15890575258@163.com (D.Y.); 13102008591@163.com (J.G.); 2 Gansu Key Laboratory of Herbivorous Animal Biotechnology, Faculty of Animal Science and Technology, Gansu Agricultural University, Lanzhou 730070, China; zhaofangfang@gsau.edu.cn; 3 College of Animal Science and Technology, Henan Agricultural University, Zhengzhou 450046, China

**Keywords:** lipid metabolism, miRNA, molecular regulation, mRNA, sheep, tail fat deposition

## Abstract

Tail fat deposition constitutes a distinctive adaptive phenotype in sheep. The Large-tailed Han (LTH) and Small-tailed Han (STH) breeds display pronounced divergence in tail fat storage, offering an ideal model for elucidating lipid metabolism regulation. Integrated sRNA-Seq and RNA-Seq analysis identified 521 differentially expressed genes and 144 miRNAs, which were significantly enriched in lipid metabolism pathways, including fatty acid metabolism and PPAR signaling. Key candidate genes (*ADIRF*, *LPL*, *ACSL5*) were highlighted as pivotal regulators. Additionally, 23 miRNA-mRNA regulatory networks were constructed, revealing complex interaction patterns from ‘one-to-one’ to ‘many-to-one’ relationships. For example, six miRNAs collectively targeted *LTA4H*. Collectively, this study advances understanding of the genetic network underlying tail fat deposition and provides candidate targets for modulating lipid metabolism. Functional validation is warranted to delineate causal mechanisms.

## Simple Summary

Fat tail development in sheep shows considerable phenotypic variation, but the genetic mechanisms remain unclear. To address this, we compared Large-tailed Han and Small-tailed Han sheep, which differ markedly in tail fat storage capacity. Transcriptomic profiling of tail adipose tissues identified 521 differentially expressed genes and 144 microRNAs. Functional enrichment analysis revealed significant involvement of lipid metabolism pathways. Further analysis pinpointed core genes regulating lipid synthesis and catabolism, and we constructed 23 miRNA–mRNA regulatory networks that showed coordinated regulation of fat storage-related genes by multiple miRNAs. These findings advance our understanding of the genetic mechanisms governing tail fat development and provide a foundation for future breeding strategies.

## 1. Introduction

Sheep are one of the most widely raised livestock species worldwide, with China as a major producer. Indigenous Chinese sheep breeds exhibit substantial phenotypic diversity, shaped by diverse climates, complex geography, and long-term natural and artificial selection [1,2]. Based on tail morphology, five types are recognized: short-fat-tailed (e.g., Mongolian, Small-tailed Han), short-thin-tailed (e.g., Tibetan), fat-rumped (e.g., Kazakh), long-fat-tailed (e.g., Large-tailed Han), and long-thin-tailed (e.g., Texel, Europe) [3,4]. The fat-tailed phenotype is considered a domestication, as ancestral wild sheep possessed thin tails [5,6,7]. Adipose tissue plays a critical role in energy homeostasis and systemic metabolism [8], functioning as both a lipid storage depot and an endocrine organ that regulates diverse physiological processes [9,10]. In sheep, tail adipose tissue shows breed-specific characteristics, with pronounced differences in fat deposition patterns between breeds such as Large-tailed Han sheep (LTH) and Small-tailed Han sheep (STH) [7]. Analogous variation occurs in pigs [11] and cattle [12]. These phenotypic differences impact economically important traits (e.g., meat quality, disease resistance) and provide valuable models for investigating the genetic and molecular mechanisms of lipid metabolism [13,14,15].

Lipid metabolism is a complex physiological process orchestrated by multiple genes. *ADIRF*, *HSD17B12*, and *LPL* play fundamental roles in lipid storage by regulating adipogenesis, catalyzing fatty acid elongation, and hydrolyzing triglycerides, respectively [16,17,18]. Concurrently, *APOBR* mediates lipoprotein uptake, *INSIG1* governs cholesterol synthesis feedback, and *THRSP* responds to nutritional cues, thereby cooperatively maintaining metabolic homeostasis [19,20,21]. Furthermore, the hub genes *ACSL5*, *FAAH*, and *ACSS2* drive lipogenic pathways via fatty acid activation, endocannabinoid degradation, and acetate utilization, respectively [22,23,24]. The functional diversity and intricate interplay of these genes collectively modulate adipose tissue deposition and metabolic homeostasis.

Recent advances in high-throughput sequencing have established small RNA (sRNA) sequencing and RNA-sequencing (RNA-seq) as powerful tools for dissecting gene regulatory and metabolic networks [25,26,27]. Among sRNAs, microRNAs (miRNAs) are critical regulators of gene expression, cell differentiation, and metabolism [28,29]. miRNAs modulate cellular functions through interactions with messenger RNAs (mRNAs), leading to translational inhibition or degradation [30]. Although numerous studies underscore the importance of miRNAs in adipocyte differentiation and lipid metabolism, most have focused on model organisms or non-tail-specific adipose depots [31,32,33,34,35,36]. Consequently, the species- and tissue-specific regulatory mechanisms governing ovine tail adipose tissue remain poorly understood. Furthermore, while transcriptome analyses have identified key adipogenesis-related genes, integrated mRNA and miRNA analyses remain scarce, limiting understanding of the synergistic molecular regulation of fat accumulation.

This study leveraged the pronounced tail phenotypic differences between LTH and STH sheep. We integrated high-throughput small RNA sequencing (sRNA-Seq) and transcriptome sequencing (RNA-Seq) data from tail adipose tissue to elucidate the genetic basis of fat deposition. Systematic analysis of mRNA and miRNA expression profiles identified differentially expressed genes (DEGs) and differentially expressed miRNAs (DE miRNAs), particularly those related to lipid metabolism. We performed functional annotation and pathway enrichment analyses to investigate the biological roles of these molecules. Additionally, a miRNA–mRNA regulatory network was constructed to reveal key regulatory functions in ovine tail fat metabolism. This research elucidated valuable resources for understanding the genetic networks governing tail fat deposition and energy metabolism in sheep. The key DEGs, DE miRNAs, and regulatory networks identified herein provide novel insights into adipose tissue biology and may yield molecular targets for optimizing fat-related traits in livestock breeding.

## 2. Materials and Methods

### 2.1. Animals and Sample Collection

**Figure 1:**
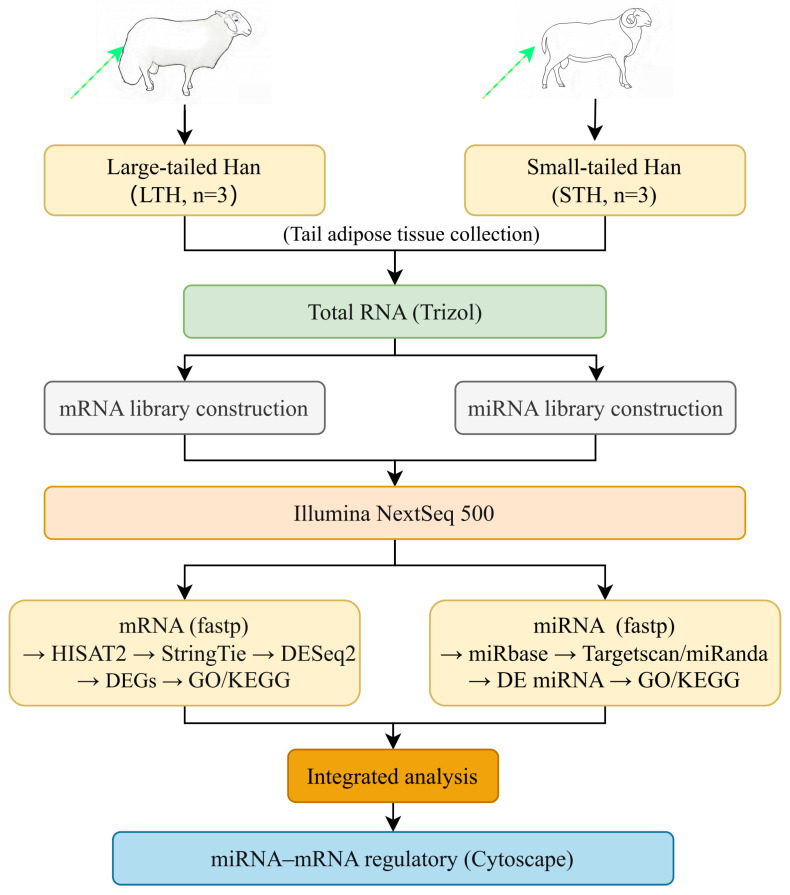
Schematic overview of the experimental design and bioinformatics analysis pipeline.

The experimental cohort comprised six healthy adult rams from two Chinese breeds: three LTHs and three STHs. Each ram served as an independent biological replicate (*n* = 3 per breed). All rams were approximately two years old, with comparable body weights (LTH: 77.49 ± 7.72 kg; STH: 78.40 ± 5.02 kg; *p* = 0.63) and had no common ancestors within three generations. Animals had ad libitum access to standardized pelleted feed and water. At the end of the experiment, rams were humanely euthanized in accordance with guidelines approved by the Institutional Animal Care and Use Committee (IACUC). Tail fat tissue samples were aseptically collected from the root of the tail, snap-frozen in liquid nitrogen, and stored at −80 °C for subsequent RNA extraction. The experimental and analytical workflow is shown in Figure 1.

### 2.2. RNA Isolation, Library Preparation, and Sequencing

Frozen tail fat tissue from each biological replicate (individual ram) was pulverized in liquid nitrogen using a pestle and mortar. Total RNA was extracted using TRIzol reagent (Invitrogen, Carlsbad, CA, USA) according to the manufacturer’s protocol. RNA integrity and concentration were assessed on an Agilent 2100 Bioanalyzer (Agilent Technologies, Palo Alto, CA, USA) with an RNA 6000 Nano kit (Agilent Technologies, Santa Clara, CA, USA) and by agarose gel electrophoresis. High-quality RNA was used to construct both miRNA and mRNA sequencing libraries. For mRNA libraries, mRNA was isolated using oligo-dT beads, thermally fragmented, and converted into double-stranded cDNA with an Illumina TruSeq RNA (Illumina, San Diego, CA, USA) to generate 2 × 150 bp paired-end reads. miRNA libraries were constructed using the Illumina TruSeq Small RNA Library Prep Kit (Illumina, San Diego, CA, USA). cDNA library quality was verified on an Agilent 2100 Bioanalyzer. miRNA sequencing was conducted on an Illumina NextSeq500 (Illumina, San Diego, CA, USA). Raw reads were generated using Illumina SCS v2.8 and RTA v1.8.70 software. High-throughput sequencing and major bioinformatic analyses were performed by PersonalBio (Shanghai, China).

### 2.3. Data Processing, Alignment, and Differential Expression Analysis

Raw sequencing reads were processed using fastp (v0.20.0) [37] to remove low-quality reads, adapter-contaminated reads, and reads containing excessive ambiguous bases (N), yielding high-quality cleaned data. For mRNA analysis, clean reads were aligned to the sheep reference genome (Oar_v3.1) using HISAT2 (v2.1.0) [38], requiring a mapping rate > 70% to ensure analytical reliability. Transcript structures were then assembled de novo using StringTie (v2.1.3) [39] to reconstruct known transcripts and predict novel isoforms. Gene expression levels were quantified as FPKM. Differential expression analysis was conducted with DESeq2 (v1.38.3) [40], with significance thresholds set at |log_2_ fold change| ≥ 0.5 and adjusted *p*-value < 0.05.

The miRNA analysis pipeline was as follows. High-quality raw reads were processed by removing 3′-adapter sequences and filtering low-quality bases, retaining only small RNA fragments of 18–26 nt. Known miRNAs were identified by aligning sequences to miRBase (v22.1) [41] using miRDeep2 (v2.0.1.2) [42]. Unannotated sequences were subsequently used for de novo prediction of novel miRNAs with MIREAP [43]. For target prediction of differentially expressed miRNAs, we combined TargetScan (v7.2) and miRanda (v3.3a) [44], retaining only the intersection as the final target gene set.

### 2.4. Differentially Expressed miRNA-Target Gene Regulatory Network Construction

A regulatory network was constructed to visualize interactions between the DE miRNA and their predicted target genes. To enhance biological relevance, the network was specifically based on the targeting relationships where a predicted target of a DE miRNA was also identified as a differentially expressed gene (DEG) in our mRNA-seq data (i.e., overlapping DEGs). This integrated network was visualized using Cytoscape (v3.7.1) [45].

### 2.5. Gene Enrichment Analysis

GO functional enrichment and KEGG pathway analyses were performed separately on two distinct gene sets: all identified DEGs and the pooled set of predicted target genes for all DE miRNAs. Both analyses were conducted using the topGO (v2.50.0) and clusterProfiler R package (v4.0.0). Terms and pathways with an adjusted *p*-value (FDR) < 0.05 were considered statistically significant.

### 2.6. RT-qPCR Analysis

To validate the reliability of our RNA-Seq results, we selected 12 DE mRNAs and 8 DE miRNAs (see Table 1 and Table 2, respectively) for experimental validation by RT-qPCR. The selected genes covered a range of expression levels to ensure the representativeness of the validation. PCR primers were designed using DNAMAN 6.0 software. For miRNA reverse transcription, we employed a poly(A) tailing method (Sangon Biotech, Shanghai, China), with *U6* snRNA as the endogenous control. mRNA was reverse-transcribed using the PrimeScript™ RT Reagent Kit (TaKaRa, Dalian, China) with ovine *β-actin* as the reference gene. All RT-qPCR amplifications were performed using SYBR Green PCR Master Mix (TaKaRa, Dalian, China). Relative expression levels of miRNA and mRNA were calculated using the 2^−(∆∆Ct)^ method. Data are presented as the mean ± standard deviation (SD). Statistical analyses were performed using SPSS 27 software. Differences between groups were assessed by *t*-test, and significance levels are denoted as follows: ns, not significant (*p* > 0.05); * *p* < 0.05; ** *p* < 0.01; and *** *p* < 0.001. Graphs were generated using GraphPad Prism 9.5.

## 3. Results

### 3.1. Sequencing Data Quality Control

RNA sequencing of tail tissue samples generated approximately 44.2 million and 43.4 million raw reads for the LTH (D) and STH (H) groups, respectively (Table 3). After quality control (QC), including the removal of low-quality bases and adapter sequences, 44.1 million (LTH) and 43.3 million (STH) high-quality clean reads were retained. These clean reads were then aligned to the sheep reference genome (*Ovis aries* assembly Oar_v3.1), achieving mapping rates of 87.61% and 85.02% for the LTH(D) and STH(H) groups, respectively. The sequencing depth and genome coverage were sufficient for downstream analysis. For sRNA sequencing, we obtained 16.6 million and 16.0 million raw reads for the LTH and STH groups. Following QC, 14.8 million and 13.5 million clean reads were obtained, respectively.

### 3.2. Analysis of DE Genes and DE miRNAs

**Figure 2:**
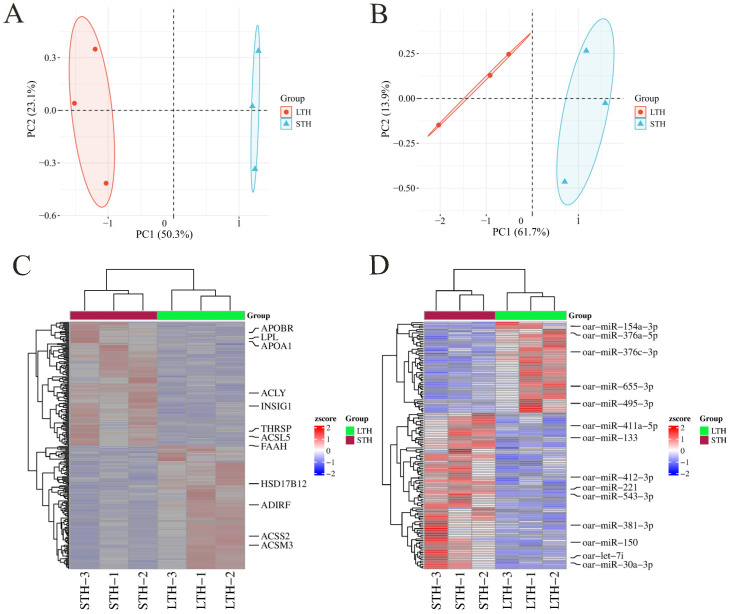
Comparative analysis of mRNA and miRNA expression profiles in LTH and STH sheep tail fat. (**A**) Principal component analysis (PCA) of mRNA expression profiles shows clear separation between the LTH and STH groups. (**B**) PCA of miRNA expression profiles similarly distinguishes the two groups. (**C**) Heatmap of differentially expressed mRNAs (DE mRNAs) between LTH and STH groups. Rows represent individual genes, and the color scale indicates expression levels (blue, downregulated; red, upregulated). (**D**) Heatmap of differentially expressed miRNAs (DE miRNAs), presented as in (**C**).

Comparative transcriptomic analysis revealed distinct expression profiles between the two breeds. Unsupervised hierarchical clustering of all expressed genes clearly separated the LTH and STH samples (Figure 2A). We identified a total of 521 DEGs between LTH and STH tail fat, with 237 upregulated and 284 downregulated in the LTH group (Figure 2C). Notably, this set of DEGs was enriched for genes with established functions in lipid metabolism, including *ADIRF*, *HSD17B12*, *ACLY*, and *LPL* (involved in fatty acid synthesis and hydrolysis); *APOBR*, *INSIG1*, and *THRSP* (core metabolic regulators); and *ACSL5*, *FAAH*, *ACSM3*, *APOA1* and *ACSS2* (implicated in lipid activation and metabolic pathways).

At the miRNA level, principal component analysis (PCA) showed clear separation of the LTH and STH groups based on their global miRNA expression patterns (Figure 2B). Sequencing identified 144 mature miRNAs across all samples. Of these, 130 (90.3%) corresponded to known sheep miRNAs annotated in miRBase (v22.1), while 14 (9.7%) were predicted as novel candidates. Differential expression analysis identified 14 significantly differentially expressed miRNAs (DEMs), with 6 upregulated and 8 downregulated in LTH compared to STH sheep (Figure 2D). Among these, six miRNAs (oar-miR-381-3p, oar-miR-376c-3p, oar-miR-154a-3p, oar-miR-655-3p, oar-miR-376a-5p, and oar-miR-495-3p) were upregulated, while eight (oar-miR-150, oar-miR-133, oar-miR-221, oar-let-7i, oar-miR-30a-3p, oar-miR-411a-5p, oar-miR-543-3p, and oar-miR-412-3p) were downregulated.

### 3.3. mRNA Functional Enrichment Analysis

**Figure 3:**
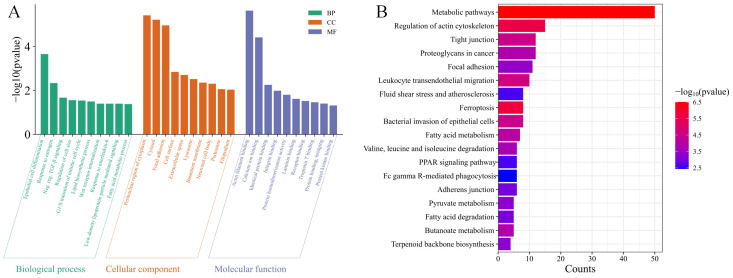
Enrichment analysis of differentially expressed mRNAs. (**A**) Gene Ontology (GO) enrichment results, showing top terms related to lipid biology and cellular structure. (**B**) KEGG pathway enrichment results, highlighting pathways in lipid metabolism and cell adhesion.

Gene Ontology (GO) enrichment analysis of differentially expressed mRNAs was performed using topGO, with a significance threshold of *p* < 0.05. The most significantly enriched terms across the three main GO categories—Biological Process, Cellular Component, and Molecular Function—are shown in Figure 3A. In the Biological Process category, genes were predominantly enriched for processes related to lipid biosynthesis and metabolism, regulation of cell proliferation and differentiation, and cellular response to estrogen and TGF-β signaling. The Cellular Component analysis highlighted significant localization to the cell periphery, cytosol, focal adhesions, and the cell surface. For Molecular Function, significant enrichments were identified for actin filament binding, calcium ion binding, and various protein–protein interaction domains.

Complementing the GO results, KEGG pathway analysis identified significant enrichment in two key functional clusters (Figure 3B). The first cluster centered on lipid metabolic processes, including fatty acid metabolism and the PPAR signaling pathway. The second cluster comprised pathways related to cell adhesion and cytoskeletal organization, such as focal adhesion and tight junction. This integrated profile suggests that tail fat accumulation involves not only metabolic reprogramming but also concomitant restructuring of the cellular microenvironment.

### 3.4. miRNA Functional Enrichment Analysis

**Figure 4:**
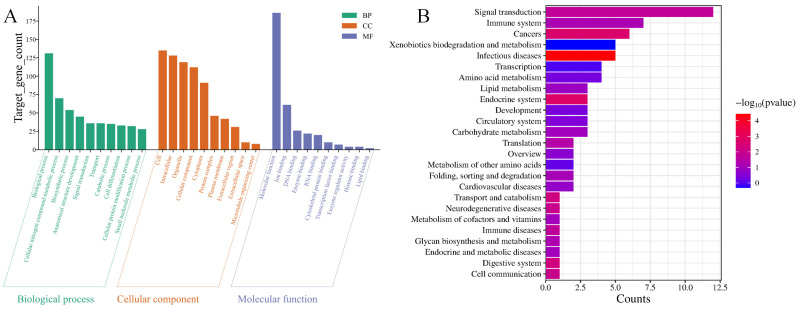
Enrichment analysis of differentially expressed miRNAs. (**A**) Gene Ontology (GO) enrichment analysis. (**B**) Kyoto Encyclopedia of Genes and Genomes (KEGG) pathway enrichment analysis.

Gene Ontology (GO) enrichment analysis of predicted target genes of the differentially expressed miRNAs was performed using topGO (v2.50.0) (significance threshold: *p* < 0.05). Significantly enriched terms across the three main GO categories are presented in Figure 4A. Within Biological Processes, target genes were enriched for terms related to cellular nitrogen compound metabolism, anatomical structure development, signal transduction, and cell differentiation. Cellular Component analysis revealed that these genes are predominantly localized to intracellular-membrane-bounded organelles, cytoplasmic regions, and protein-containing complexes. For Molecular Function, significant enrichments were identified for ion binding (e.g., metal ion), nucleic acid binding (DNA/RNA), and enzyme binding activities.

KEGG pathway analysis revealed that the target genes of these miRNAs were significantly enriched in specific pathways (Figure 4B). These included pathways involved in signal transduction, immune response, xenobiotic biodegradation and metabolism, and cancer. Significant enrichment was also detected in core metabolic pathways, such as those for lipid and amino acid metabolism. Collectively, these results imply that the miRNAs may participate in regulating key biological processes, including cellular metabolism, signal transduction, immune-metabolic crosstalk, and pathways linked to disease.

### 3.5. Construction of the miRNA–mRNA Interaction Network

**Figure 5:**
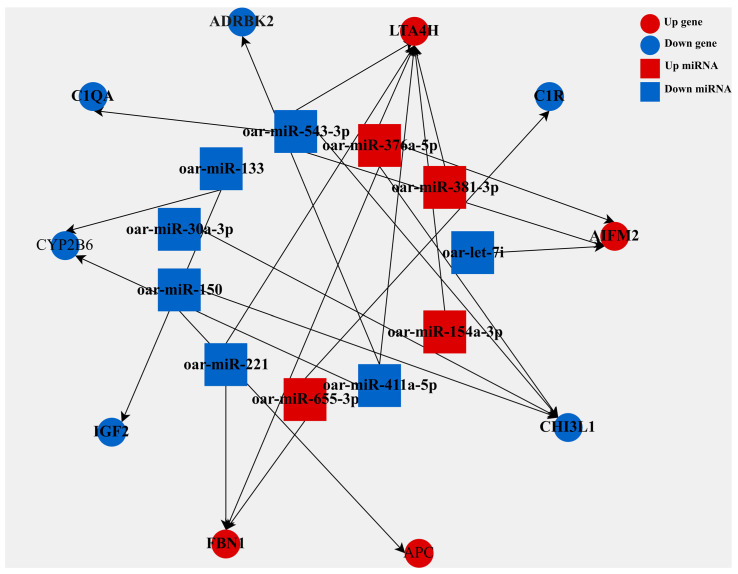
Regulatory network of differentially expressed miRNAs and their predicted target mRNAs. miRNA and mRNA nodes are depicted as squares and circles, colored blue (downregulated) and red (upregulated).

To elucidate the regulatory network involving miRNAs and mRNAs in lipid metabolism, we integrated miRNA target prediction with transcriptomic sequencing data. This analysis identified 23 significantly differentially expressed miRNA-mRNA regulatory pairs, comprising 11 miRNAs and 10 target genes (Figure 5). Notably, 9 pairs (39.1%) exhibited a negative correlation, consistent with the canonical repressive function of miRNAs in post-transcriptional regulation. Within this network, *LTA4H* was identified as the key hub gene, being targeted by six miRNAs (oar-miR-154a-3p, -221, -376a-5p, -381-3p, -411a-5p, and -543-3p). Among these interactions, three were negative regulatory pairs. *CHI3L1* was targeted by four miRNAs, while *AIFM2* and *FBN1* were each targeted by three. From the miRNA perspective, oar-miR-376a-5p and oar-miR-543-3p each regulated four target genes, representing the broadest targeting range, followed by oar-miR-411a-5p (three targets). Focusing on the negative regulatory subset, oar-miR-543-3p and oar-miR-221 were involved in the highest number of negatively correlated pairs.

### 3.6. Validation of mRNA and miRNA Expression by RT-qPCR

**Figure 6:**
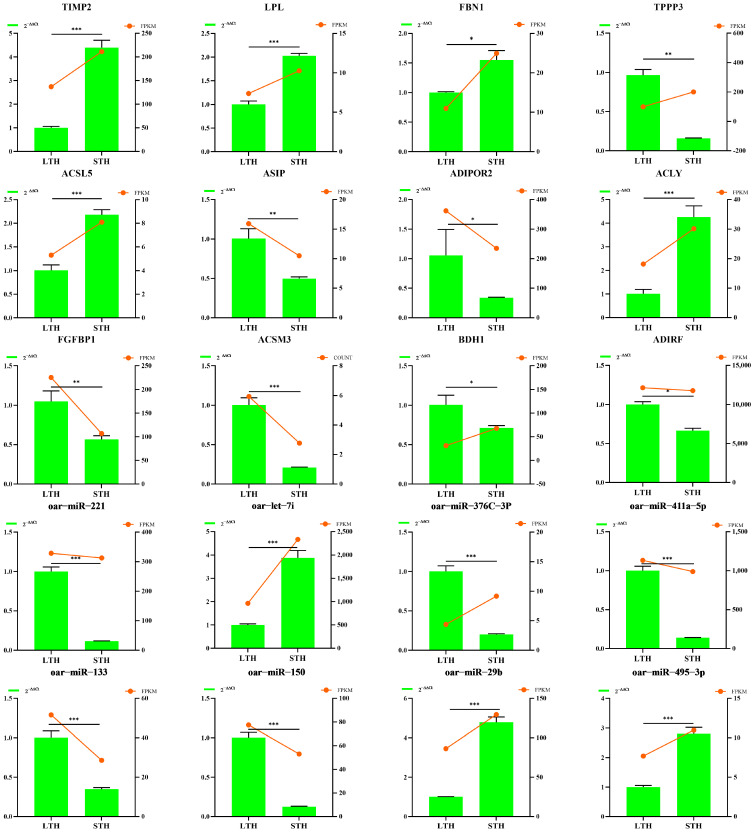
Validation of RNA-Seq results by RT-qPCR. Expression levels of selected mRNAs and miRNAs measured by RNA-Seq (in FPKM) and RT-qPCR (quantified using the 2^−∆∆Ct^ method) are compared. Differences between groups were assessed by Student’s *t*-test. Data are presented as mean ± SD. Significance levels are denoted as: ns, not significant (*p* > 0.05); * *p* < 0.05; ** *p* < 0.01; and *** *p* < 0.001.

To validate the sequencing data, we performed RT-qPCR on twelve selected mRNAs and eight miRNAs. The results showed a high agreement between the two methods, with consistency rates of 83.3% for mRNAs and 87.5% for miRNAs, confirming the reliability of the sequencing data (Figure 6).

## 4. Discussion

The distribution of adipose tissue in mammals is governed by a combination of genetic factors, central nervous system signaling, and dynamic changes in the gut microbiota, with genetic predisposition being the primary determinant [46]. The Large-tailed Han sheep (LTH), a breed with a distinct fat-tail phenotype, exhibits substantial adipocyte accumulation specifically in the caudal region [47,48]. Compared to other adipose depots, tail fat demonstrates unique developmental traits, such as delayed maturation, pronounced adipocyte hypertrophy, and distinct metabolic activity [49,50]. Adipose tissue expansion in this depot occurs primarily through lipid-laden adipocyte hypertrophy, a process driven by the mechanisms of fatty acid storage and mobilization [51]. However, the precise genetic and molecular mechanisms underlying this specialized fat deposition in fat-tailed sheep remain not fully elucidated. To address this gap, we performed comparative transcriptomic analyses of mRNA and miRNA expression in the tail adipose tissue of LTH and STH sheep.

The fat-tail phenotype is an important adaptive trait in sheep (*Ovis aries*), resulting from domestication for extreme climate tolerance and exhibiting notable breed-specific genetic regulation. Unlike intramuscular fat in cattle or backfat in pigs, ovine tail adipose tissue is distinct in its developmental timing, metabolic activity, and miRNA expression profile [52,53]. In this study, we identified 144 conserved miRNAs in sheep tail fat, a number considerably lower than the >3000 miRNAs often reported [54]. This disparity likely stems from differences in sequencing depth, bioinformatic pipelines for miRNA identification, and the inherent biological variation in miRNA expression across breeds and adipose depots. Differential expression analysis revealed 521 DEGs between LTH and STH tail fat tissues. Although the number of DEGs aligns with previous studies on ovine adipose tissue [55,56], the specific genes identified show limited overlap, reinforcing the hypothesis that the transcriptional regulation of tail fat deposition is highly breed-specific.

Our sequencing analysis revealed significant transcriptional differences in key genes implicated in lipid metabolism and adipocyte biology, including *ADIRF*, *HSD17B12*, *LPL*, *APOBR*, *INSIG1*, *THRSP*, *ACSL5*, *FAAH*, and *ACSS2*. This pattern suggests a concerted regulatory shift in lipid handling within the tail fat depot. Notably, several of these genes are established players in adipogenesis and lipid homeostasis: *ACLY* [57], *ACSS2* [58], and *THRSP* [59] are involved in fatty acid synthesis; *LPL* [60] hydrolyzes triglycerides to provide free fatty acids for storage; and *INSIG1* [61] regulates the *SREBP* pathway, a master regulator of lipogenesis. Furthermore, *APOA1* [62] and *APOBR* [19] function in lipoprotein metabolism and uptake, while *ADIRF* [63] and *HSD17B12* [64] may modulate the local adipogenic and hormonal microenvironment. Of particular interest is *FGFBP1*, a gene identified in our study whose role in ovine tail fat has been confirmed. In adipocytes, *FGFBP1* overexpression inhibits proliferation and promotes differentiation, whereas its silencing has opposite effects [65]. This positions *FGFBP1* as a potential regulator of the preadipocyte fate decision—shifting cells from proliferation to lipid storage—which could be a critical mechanism governing tail fat expansion. However, the specific functions and interactions of these genes within the unique context of ovine tail fat biology remain to be experimentally validated. Future cellular and functional studies are needed to confirm their precise roles.

This study aimed to construct a potential miRNA–mRNA regulatory network associated with adipogenesis in sheep. Several DE miRNAs are predicted to interact with lipid metabolism-related DEGs, suggesting their coordinated regulatory roles. The let-7 family represents key regulators of glucose homeostasis [66]. In tail fat, we identified two synergistic regulatory interactions—oar-let-7i–*AIFM2* and oar-miR-543-3p–*AIFM2*—which we hypothesize facilitate lipid storage via a dual mechanism: (1) downregulation of oar-let-7i attenuates its inhibition of insulin signaling pathways, thereby enhancing glucose uptake, while (2) concurrently relieving post-transcriptional repression of the ferroptosis inhibitor *AIFM2*. *AIFM2* upregulation promotes adipocyte survival by enhancing mitochondrial antioxidant capacity rather than activating lipolysis-related apoptotic pathways [67]. As *AIFM2* is also a predicted target of oar-miR-543-3p, the concurrent downregulation of both miRNAs may synergistically enhance adipocyte antioxidant capacity by derepressing *AIFM2*, thereby providing a more stable cellular environment conducive to lipid storage. We also identified the oar-miR-221–*FBN1* and oar-miR-221–*LTA4H* regulatory networks. *FBN1* is expressed during adipogenesis [68], and its level positively correlates with adipose tissue mass, suggesting a role in the structural expansion of adipocytes. *LTA4H* is a key enzyme synthesizing the inflammatory mediator leukotriene B4 and is involved in inflammatory metabolic processes [69]. Although the direct role of oar-miR-221 in adipogenesis remains elusive, our sequencing analyses suggest that its downregulation may derepress extracellular matrix proteins such as *FBN1* and metabolic enzymes including *LTA4H*, thereby cooperatively shaping a microenvironment conducive to adipocyte maturation and lipid storage. In future studies, we plan to validate the aforementioned predicted targeting relationships through functional experiments, such as dual-luciferase reporter assays, miRNA overexpression, or knockdown. Furthermore, elucidating the specific roles of key target genes in adipocyte ferroptosis and lipid metabolism will facilitate a more comprehensive understanding of the molecular mechanisms underlying excessive tail fat deposition—a unique trait in sheep—and may yield novel insights into the regulation of related metabolic diseases.

## 5. Conclusions

Through transcriptomic and miRNA analysis of sheep tail adipose tissue, this study identified 521 DEGs and 144 DE miRNAs between Large-tailed and Small-tailed Han sheep. These molecules were significantly enriched in pathways associated with lipid metabolism, particularly fatty acid metabolism and the PPAR signaling pathway. An integrated analysis further revealed 23 potential miRNA-mRNA regulatory pairs. These findings refine our understanding of the transcriptional regulation underlying tail fat deposition and offer new insights into the genetic determinants of ovine adipose traits. While experimental validation remains necessary, the identified lipid metabolism genes and miRNA–mRNA networks provide a prioritized set of candidate targets for future mechanistic studies and for the precision breeding of fat-tail phenotypes in sheep.

## Figures and Tables

**Table 1:** mRNA primer sequence.

Gene	Forward (5′ → 3′)	Reverse (5′ → 3′)
*TIMP2*	TGGCAACGACATCTATGGCAACC	TCCTCCAATGTCCAGCGAGACC
*LPL*	CGCCGCCGACAGGATTACAAG	CTCCAGGAATGAGGTGGCAAGTG
*FBN1*	GAGCCTTGCCAACCATGTCCTTC	ATCGCTGCCTGCTGACGTTATTC
*TPPP3*	AGTACACGGGCTCCCACAAGG	GCACTCACGTAGCCACTGTCATC
*ACSL5*	CAGTTCAGTCGCTCAGTCGTGTC	ACCGAGGATGAGATGGCTGGATG
*ASIP*	TGGATGTCAGCCGCCTCTTCC	GCTTTTCCTCAGGTGCCAGGTG
*ADIPOR2*	AGGCTCAGGAAAGGGCACCAG	GCTCCATCATCGCACTCACACTC
*ACLY*	TCACCGAGGTCTTCAAGGAGGAG	GCTGTCACCATCAGGCACATCTC
*FGFBP1*	CCTCCTCCTTCTGGCTGTTCTG	TGCTTGGTTGGCTGGCTCCT
*ACSM3*	TCTGACACAGGCTGGGCAAAATC	AAGATGGAAGCTGGCTCAAACCG
*BDH1*	AAGGTCGCCAGGATGGAGAGC	TCCGCAGCCACCAGTAGTAGTC
*ADIRF*	CCACAGAAGCAGGGCAGAAAGC	CCCAAAACCCGAGAAAGCCTCAG
*β-actin*	GCAGGTCATCACCATCGGCAAT	CGTGTTGGCGTAGAGGTCCTTG

**Table 2:** miRNA primer sequence.

miRNA	Forward (5′ → 3′)
oar-miRNA-221	CCGAGCTACATTGTCTGCTGGGT
oar-miRNA-376C-3p	CGCCAACATAGAGGAAATTCCACGT
oar-miRNA-411a-5p	GCGATAGTAGACCGTATAGCGTACG
oar-miRNA-133	ATATTGGTCCCCTTCAACCAGCTGT
oar-miRNA-150	TCTCCCAACCCTTGTACCAGTG
oar-miRNA-29b	CGGCTAGCACCATTTGAAATCAGTGT
oar-miRNA-495-3p	CGCAAACAAACATGGTGCACTTCTT
oar-let-7i	CCGTGAGGTAGTAGTTTGTGCTGTT

**Table 3:** Quality control metrics for mRNA and miRNA sequencing data.

Libraries	Sample	Average Raw Reads	Average Remaining Clean Reads	Average Mapped Reads (%)	Average Q20 Ratio	Average Q30 Ratio	Average GC Content
mRNA	LTH(D)	44,236,875	44,106,884	38,642,923 (87.61%)	96.38%	93.25%	49.20%
	STH(H)	43,442,331	43,267,929	36,786,826 (85.02%)	95.71%	92.26%	50.4%
miRNA	LTH(D)	16,634,363	14,750,176	6,545,904 (44.38%)	96.91%	91.07%	46.35%
	STH(H)	16,012,976	13,539,464	5,886,429 (43.47)	96.24%	91.2%	45.21%

## Data Availability

All data generated and/or analyzed in this study are available from the corresponding author upon reasonable request. The mRNA and miRNA sequencing data for sheep tail fat have been deposited in the Sequence Read Archive (SRA) database of NCBI, under the project IDs PRJNA1298782.and PRJNA1299042.

## Abbreviations

The following abbreviations are used in this manuscript:

LTH	Large-tailed Han sheep
STH	Small-tailed Han sheep
miRNA	MicroRNA
DEGs	Differentially expressed genes
DE miRNAs	Differentially expressed miRNAs
GO	Gene Ontology
KEGG	Kyoto Encyclopedia of Genes and Genomes
